# Simple and Rapid Synthesis of Magnetite/Hydroxyapatite Composites for Hyperthermia Treatments via a Mechanochemical Route

**DOI:** 10.3390/ijms14059365

**Published:** 2013-04-29

**Authors:** Tomohiro Iwasaki, Ryo Nakatsuka, Kenya Murase, Hiroshige Takata, Hideya Nakamura, Satoru Watano

**Affiliations:** 1 Department of Chemical Engineering, Graduate School of Engineering, Osaka Prefecture University, 1-1 Gakuen-cho, Nakaku, Sakai, Osaka 599-8531, Japan; E-Mails: rnakatsuka@chemeng.osakafu-u.ac.jp (R.N.); hnakamura@chemeng.osakafu-u.ac.jp (H.N.); watano@chemeng.osakafu-u.ac.jp (S.W.); 2 Department of Medical Physics and Engineering, Faculty of Health Science, Graduate School of Medicine, Osaka University, 1-7 Yamadaoka, Suita, Osaka 565-0871, Japan; E-Mails: murase@sahs.med.osaka-u.ac.jp (K.M.); takata@sahs.med.osaka-u.ac.jp (H.T.)

**Keywords:** magnetite, carbonate hydroxyapatite, mechanochemical effect, hyperthermia

## Abstract

This paper presents a simple method for the rapid synthesis of magnetite/hydroxyapatite composite particles. In this method, superparamagnetic magnetite nanoparticles are first synthesized by coprecipitation using ferrous chloride and ferric chloride. Immediately following the synthesis, carbonate-substituted (B-type) hydroxyapatite particles are mechanochemically synthesized by wet milling dicalcium phosphate dihydrate and calcium carbonate in a dispersed suspension of magnetite nanoparticles, during which the magnetite nanoparticles are incorporated into the hydroxyapatite matrix. We observed that the resultant magnetite/hydroxyapatite composites possessed a homogeneous dispersion of magnetite nanoparticles, characterized by an absence of large aggregates. When this material was subjected to an alternating magnetic field, the heat generated increased with increasing magnetite concentration. For a magnetite concentration of 30 mass%, a temperature increase greater than 20 K was achieved in less than 50 s. These results suggest that our composites exhibit good hyperthermia properties and are promising candidates for hyperthermia treatments.

## 1. Introduction

Hydroxyapatite (Ca_10_(PO_4_)_6_(OH)_2_; denoted hereafter as HA) is a calcium phosphate ceramic that has found wide use as a biomaterial in various applications (e.g., artificial bone and dental root construction, cosmetic foundation, *etc*.) owing to its high biocompatibility and chemical stability. Many efforts are underway to find new applications for HA functionalization by incorporating effective organic or inorganic materials, such as chitosan [1–3], collagen [4–6], gelatin [7,8], carbon nanotubes [9–11], titania [12–14], and zirconia [15,16]. In particular, magnetite-incorporated HA (Fe_3_O_4_/HA) composites have attracted much attention as a promising functional material for developing, for example, adsorbents [17–19], catalysts [20,21], and hyperthermia agents [22–24]. Recently, the possible applications of composites such as magnetic scaffolds in bone tissue engineering have been actively studied [25–27].

It is well known that Fe_3_O_4_ nanoparticles are biocompatible with the human body. However, there is concern that the nanoparticles may have long-term toxic effects. The use of magnetic Fe-doped HA is the most effective solution to this problem [28–30], but embedding Fe_3_O_4_ nanoparticles into HA matrices can also reduce the long-term toxic effects. Such Fe_3_O_4_-embedded HA materials are conventionally synthesized by mixing HA powder with Fe_3_O_4_ nanoparticles, which are prepared sequentially or individually prior to mixing [22–24,31]. However, conventional synthesis methods have some disadvantages: the reaction times required for the complete formation of HA and Fe_3_O_4_ are relatively long, and subsequent heat treatments performed to achieve aging and crystallization are required. Therefore, a simple method for rapidly fabricating Fe_3_O_4_/HA composites is needed to enable the industrial-scale production of these materials.

In this paper, we describe a method for quickly and easily synthesizing Fe_3_O_4_/HA composite particles. In the method, Fe_3_O_4_ nanoparticles are first prepared by coprecipitation [32], after which we employ a horizontal tumbling ball mill to mechanochemically synthesize submicron-sized HA particles under wet conditions. These operations are sequentially performed at room temperature and do not require any subsequent heat treatments. The merits of using horizontal tumbling ball mills are that the device structure is simple, the handling is easy, the energy consumption is relatively low, and scale-up is easily achieved [33]. The wet mechanochemical process promotes the dispersion of Fe_3_O_4_ nanoparticles in the HA matrix. We investigated the factors and conditions that influence the formation of Fe_3_O_4_/HA composites and examined their properties with respect to hyperthermia treatments.

## 2. Results and Discussion

### 2.1. Properties of Superparamagnetic Fe_3_O_4_ Nanoparticles

**Figure 1:**
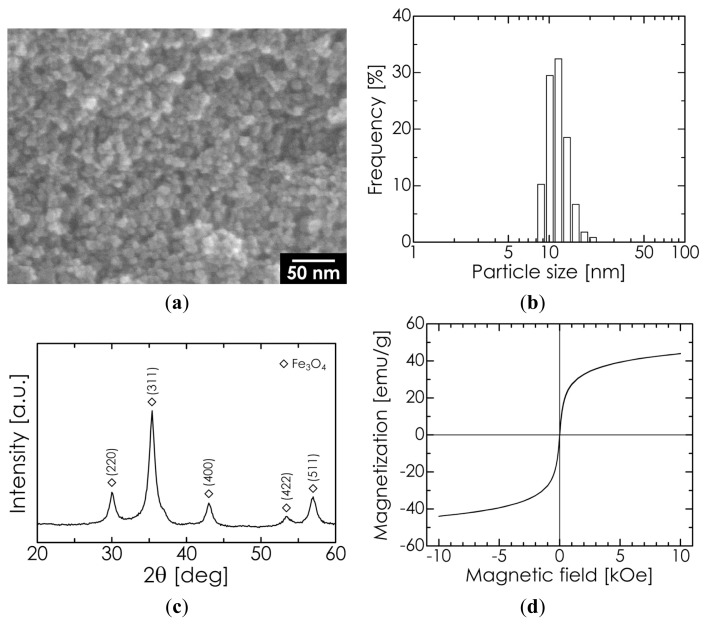
(**a**) Scanning electron microscopy (SEM) image; (**b**) dynamic light scattering (DLS) particle size distribution; (**c**) X-ray diffraction (XRD) pattern; and (**d**) magnetization-magnetic field hysteretic cycle of Fe_3_O_4_ nanoparticles.

For Fe_3_O_4_ nanoparticles synthesized by a coprecipitation method [32], a scanning electron microscopy (SEM) image, dynamic light scattering (DLS) particle size distribution, X-ray diffraction (XRD) pattern, and magnetization-magnetic field hysteresis cycle (measured at room temperature) are all shown in Figure 1. As shown in the SEM image, the particle diameter is approximately 10–20 nm, which is close to the median diameter (10.6 nm) of the measured DLS size distribution. The XRD pattern shows only the typical diffraction lines of Fe_3_O_4_. The lattice constant was determined to be 8.402 Å from several diffraction angles that showed high-intensity lines; indeed, the lattice constant is in close agreement with the standard value for Fe_3_O_4_ (8.396 Å), as opposed to that for maghemite (8.345 Å). These results suggest that our manufactured Fe_3_O_4_ nanoparticles were of high purity. The average crystallite size was calculated to be 10.9 nm from the full width at half-maximum (FWHM) of the Fe_3_O_4_ (311) diffraction line at 2θ = 35.5°, using Scherrer’s formula. This value matches the results of SEM observation and the measured median diameter, implying that our Fe_3_O_4_ nanoparticles possessed a single-crystal structure. The saturation magnetization was observed to be 47 emu/g, which is approximately half of the value for the corresponding bulk (92 emu/g) due to the smaller size and lower crystallinity [34]. The coercivity of the particles was determined to be 3 Oe, indicating superparamagnetism.

### 2.2. Mechanochemical Synthesis of HA

**Figure 2:**
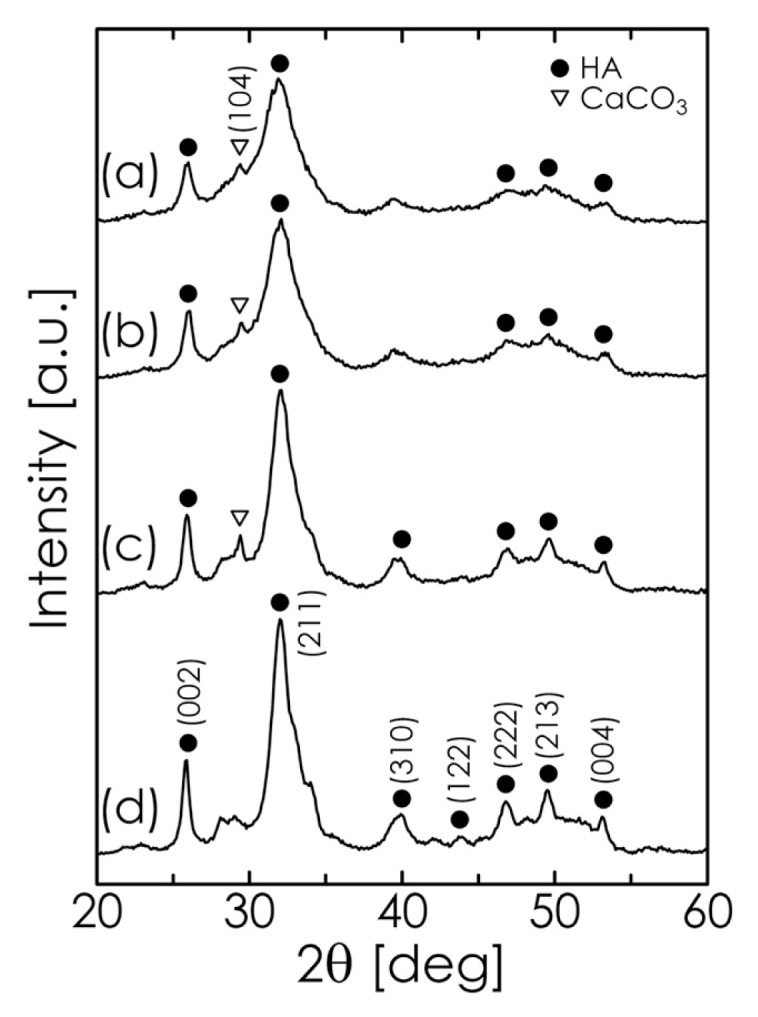
XRD pattern of samples obtained after milling for 6 h at different pH values: (**a**) 12.0; (**b**) 12.5; (**c**) 13.0; and (**d**) 13.5.

To investigate the formation of HA during the mechanochemical process, Fe_3_O_4_-free HA was first synthesized using a suspension of CaHPO_4_·2H_2_O (dicalcium phosphate dihydrate; denoted hereafter as DCPD) and CaCO_3_ as the starting material. The pH of this suspension was varied between 12.0 and 13.5 by adjusting the amount of NaOH solution added. The XRD patterns for several samples obtained by a 6-h milling process at different pH values are shown in Figure 2. When the pH was lower than 13.0, CaCO_3_ remained in the products even after 6 h. At a pH of 13.5, however, the diffraction lines corresponding to CaCO_3_ disappeared, implying that the formation reaction of HA (expressed by Equation (1) [35–37]) was complete after milling.

(1) $$6{\text{CaHPO}}_{4}\cdot 2{\text{H}}_{2}\text{O}+4{\text{CaCO}}_{3}\to {\text{Ca}}_{10}{\left({\text{PO}}_{4}\right)}_{6}{\left(\text{OH}\right)}_{2}+14{\text{H}}_{2}\text{O}+4{\text{CO}}_{2}$$

At higher pH values, the formation reaction and crystallization of HA tended to progress more rapidly even at room temperature because NaOH may prompt CO_2_ gas (generated in the process) to dissolve into the solution and allow for the stabilization of the HA phase at higher pH values [38–40].

**Figure 3:**
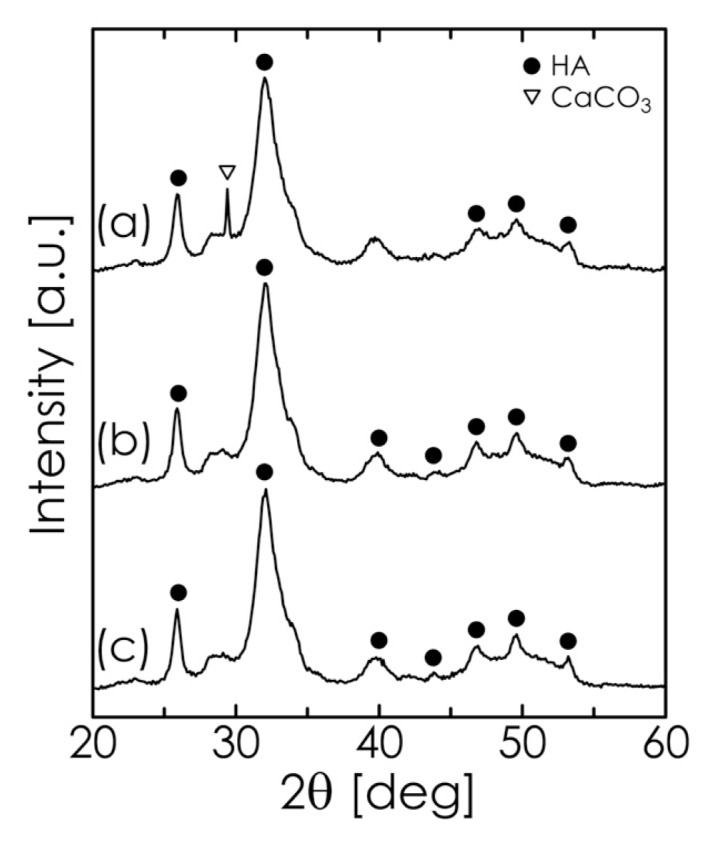
XRD pattern of samples obtained at pH 13.5 after milling for (**a**) 30 min, (**b**) 1 h, and (**c**) 2 h.

We also studied the effect of the milling time on the formation of HA while maintaining the pH at 13.5. As shown in Figure 3, DCPD (showing an intense diffraction line at 2θ = 20.9°) had already disappeared after 30 min. On the other hand, the diffraction lines indicating the presence of CaCO_3_ decreased with increasing milling time, while the characteristic reflections of HA were clearly observed. When the milling time was longer than 1 h, we obtained a single phase of HA. In addition, we confirmed that the sample obtained by vigorously stirring the starting suspension for 1 h (without milling) contained large amounts of DCPD and CaCO_3_ (data not shown). From these results, we can conclude that milling and high-pH conditions effectively contribute to the rapid formation of HA.

**Figure 4:**
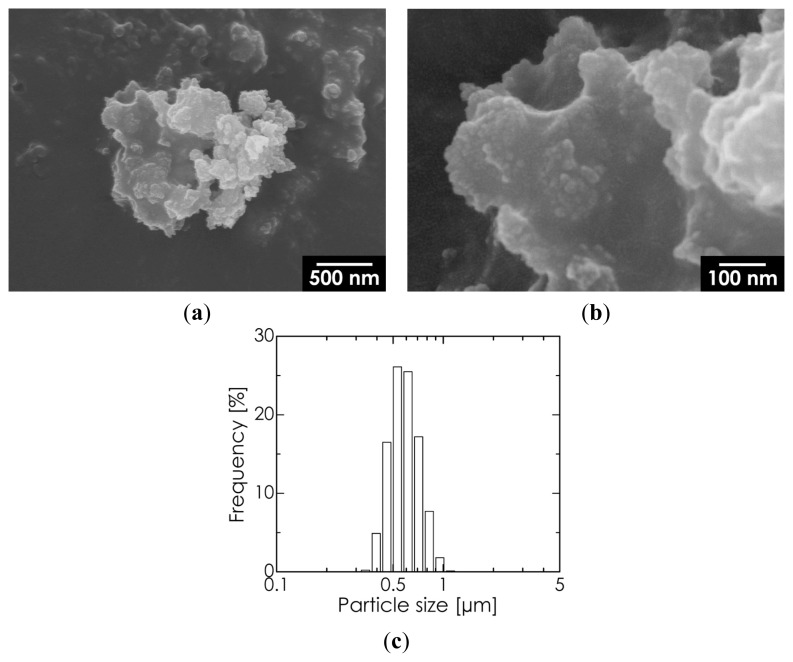
SEM images captured at (**a**) low and (**b**) high magnification and (**c**) DLS particle size distribution of the hydroxyapatite (HA) sample.

We selected an HA sample synthesized by milling for 1 h at a pH of 13.5 for further characterization. As shown in Figure 4, this HA sample consisted of aggregates measuring several micrometers, while the primary particle size was confirmed to be on the sub-micrometer scale. The Ca/P molar ratio for this sample was 1.69, determined using inductively coupled plasma-optical emission spectrophotometry (ICP-OES), which is quite close to the theoretical value (1.67).

**Figure 5:**
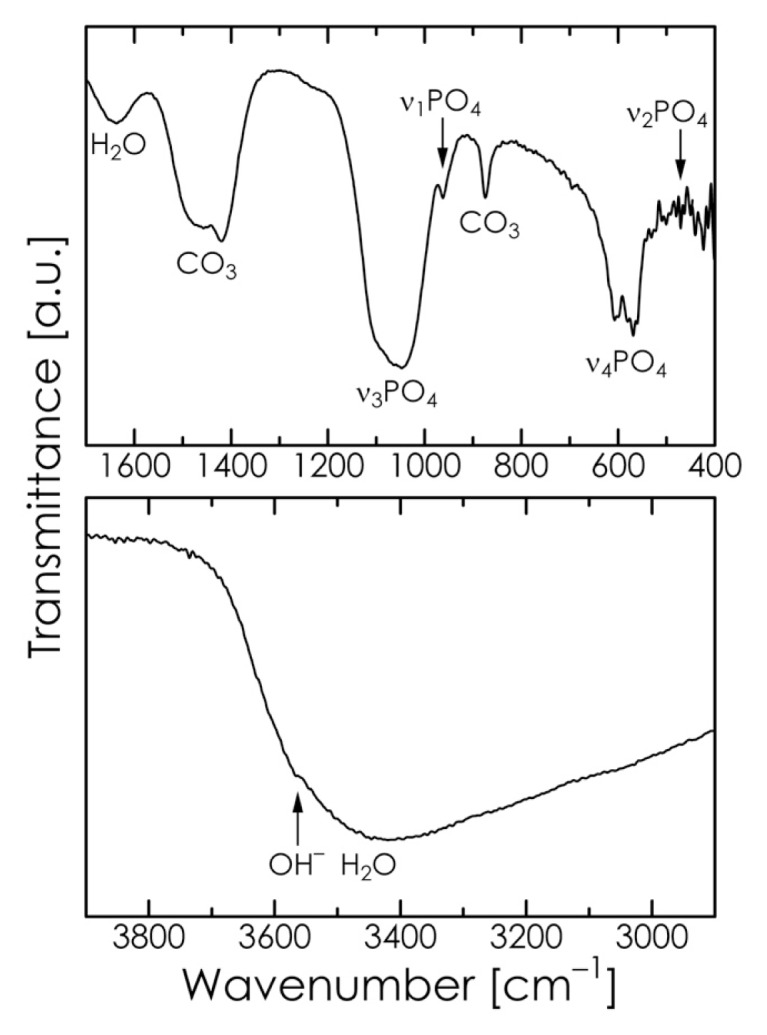
Fourier transform-infrared (FT-IR) spectrum of the HA sample.

The Fourier transform-infrared (FT-IR) spectrum of the chosen HA sample is shown in Figure 5. The absorption bands at 580, 962, and 1047 cm^−1^ and those at 471, 569, and 607 cm^−1^ are attributable to the stretching mode and bending mode of PO_4_^3−^ in HA, respectively. The peak at 1636 cm^−1^ and the broad peak at approximately 3450 cm^−1^ correspond to the water of crystallization present in the sample. The weak absorption band at approximately 3560 cm^−1^ can be attributed to the stretching vibration mode of the OH^−^ in the lattice [18,40]. The absorption bands at 875, 1419, and 1457 cm^−1^ indicate the presence of CO_3_^2−^, which suggests that carbonate ions were substituted for certain phosphate positions in the apatite lattice. The substitution may be assumed to be B-type because the HA sample did not show the typical absorption band at 1540 cm^−1^ that is found in A-type carbonate HA [39,41,42]. Our CHN elemental analysis also confirmed that the HA sample contained 9.6 mass% of carbonate ion, which is somewhat greater than that of bone apatite (6–8 mass% [43]). The carbonate content of this material may be reduced by replacing some of the CaCO_3_ used as the starting material with Ca(OH)_2_ [44].

**Figure 6:**
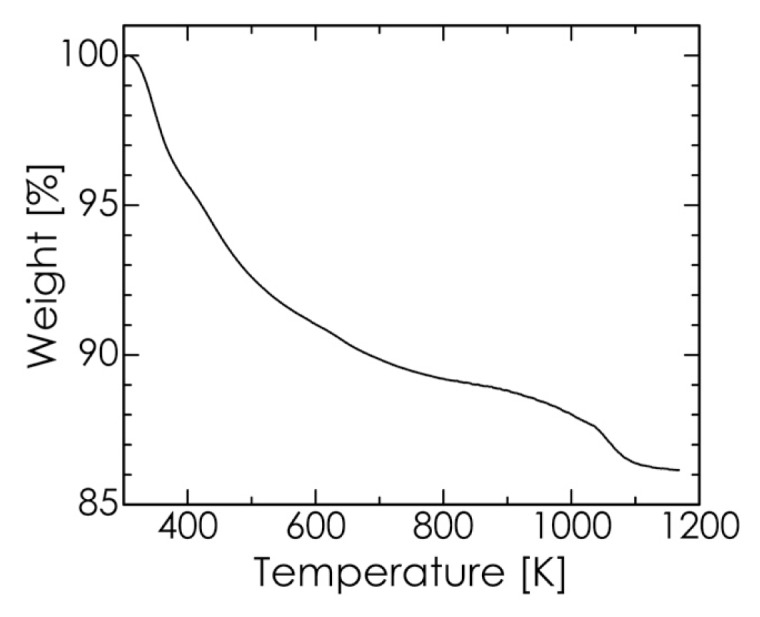
Thermogravimetric (TG) curve of the HA sample.

The thermogravimetric (TG) curve for the chosen HA sample is shown in Figure 6. The weight loss at temperatures up to approximately 500 K is attributable to the desorption of adsorbed water, whereas the additional weight loss from 600 to 800 K is due to the loss of lattice water [41]. The slight weight loss above 800 K is mainly the result of the dehydroxylation of HA and the evolution of CO_2_ [39,40,45]. In the temperature range of 650–1050 K, over which CaCO_3_ is decomposed, no rapid weight loss was observed in the TG curve. This suggests that the HA sample contained no unreacted CaCO_3_, which in turn implies that the purity of HA was high in the sample [40]. Therefore, the observed thermal decomposition behavior of our HA sample agreed well with the typical results obtained for carbonate HA as reported in the literature. Accordingly, we conclude that our proposed mechanochemical method can produce uniform B-type carbonate-substituted HA simply and rapidly without the need for any subsequent heat treatments such as aging, annealing, or sintering. The HA powder synthesized by our method has relatively low crystallinity, as shown in Figure 3, and may be quite suitable as a bone-substitute material [43].

### 2.3. Hyperthermia-Relevant Properties of Fe_3_O_4_/HA Nanocomposites

**Figure 7:**
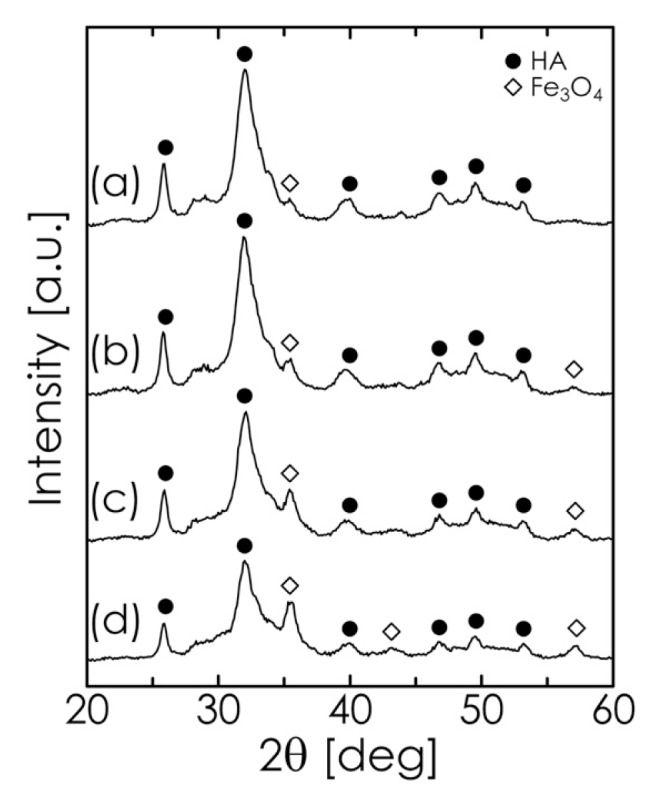
XRD pattern of Fe_3_O_4_/HA composites with different Fe_3_O_4_ concentrations: (**a**) 5; (**b**) 10; (**c**) 20; and (**d**) 30 mass%.

Based on the results described above, we synthesized Fe_3_O_4_/HA composites, where a starting suspension (consisting of Fe_3_O_4_, DCPD, and CaCO_3_) with a pH of 13.5 and a milling time of 1 h were employed. The XRD patterns of composites with different Fe_3_O_4_ concentrations are shown in Figure 7, where the diffraction intensities of Fe_3_O_4_ vary as a function of the Fe_3_O_4_ concentration. The composites were observed to consist of two phases, Fe_3_O_4_ and HA. From the DLS analysis, the median diameters of composite particles containing 5, 10, 20, and 30 mass% Fe_3_O_4_ were 0.57, 0.54, 0.53, and 0.57 μm, respectively, which were close to those of the Fe_3_O_4_-free HA particles (0.57 μm). These results indicate that Fe_3_O_4_ incorporation hardly affected the growth of the composite particles. It is difficult to transport such large particles to tissues to be treated through the vascular system. However, composite particles can be directly implanted in bone defects after the excision of tumoral bone [23].

**Figure 8:**
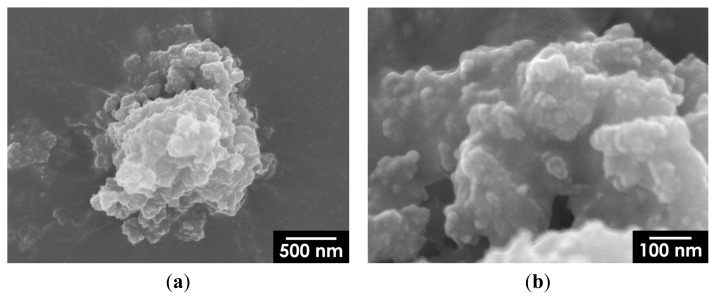
SEM images of 5 mass% Fe_3_O_4_/HA composite nanoparticles: (**a**) low- and (**b**) high-magnification views. The magnification of Figure 8b is the same as that of Figure 4b.

**Figure 9:**
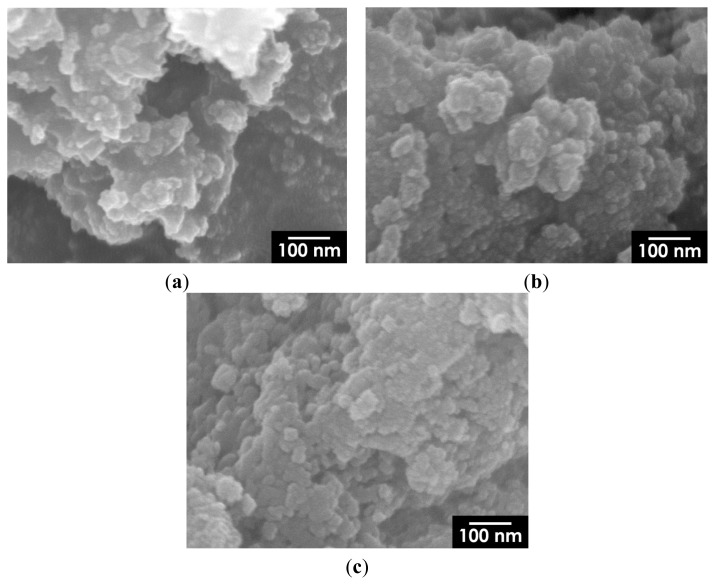
SEM images of surface of (**a**) 10 mass%; (**b**) 20 mass%; and (**c**) 30 mass% Fe_3_O_4_/HA composite particles. The magnification of all images is the same as that of Figure 8b.

A representative SEM image of composite particles containing 5 mass% Fe_3_O_4_ is shown in Figure 8. The figure shows Fe_3_O_4_ nanoparticles measuring approximately 10 nm embedded in an HA matrix; the particles are homogeneously dispersed without having formed any large aggregates. As shown in Figure 9, large isolated aggregates of Fe_3_O_4_ nanoparticles were hardly observed on the surface of HA particles, even at higher Fe_3_O_4_ concentrations; thus, we confirmed that our synthesis method effectively yields nanostructured Fe_3_O_4_/HA composites.

Ansar *et al.* [31] synthesized Fe_3_O_4_/HA composite particles with characteristics similar to those of our composites using a simple method. In their synthesis method, however, after the formation of Fe_3_O_4_ nanoparticles by coprecipitation, they employed heating treatments at 343 K for 1 h and then at 353 K for 1 h to synthesize HA. Furthermore, aging for 24 h at room temperature was required after heating. In contrast, our method requires no reaction after coprecipitation and requires only 1 h of milling at room temperature. Accordingly, our method is very simple compared to the one mentioned above, which is an advantage of our method over conventional techniques.

**Figure 10:**
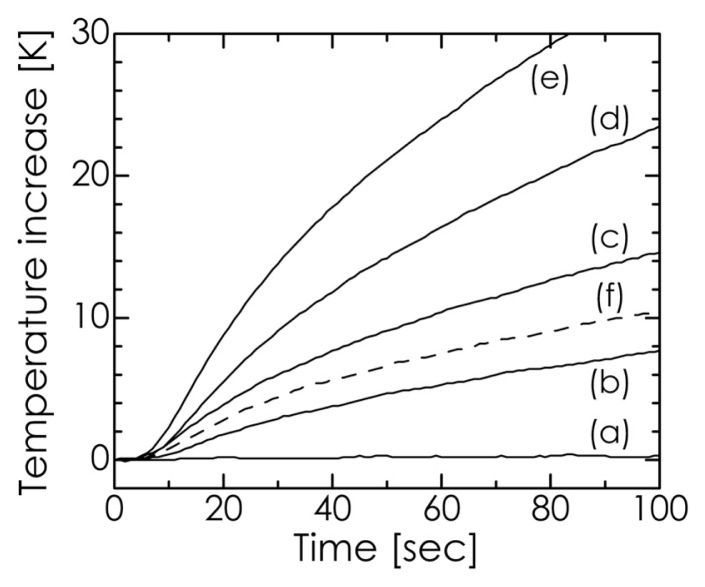
Temperature profiles of the (**a**) HA sample and Fe_3_O_4_/HA composites containing (**b**) 5, (**c**) 10, (**d**) 20, and (**e**) 30 mass% Fe_3_O_4_ in an alternating magnetic field. The broken line (**f**) indicates the result for the control experiment using the 10 mass% Fe_3_O_4_/HA mixture prepared via stirring.

The temperature increase in our composites when submitted to an alternating magnetic field is depicted in Figure 10. As the Fe_3_O_4_ concentration was increased, the temperature rose more rapidly. In particular, for a Fe_3_O_4_ concentration of 30 mass%, a temperature increase of more than 20 K was achieved over a period of less than 50 s. Furthermore, as indicated by curves c and f in Figure 10, our composite was confirmed to exhibit higher heating efficiency compared with the Fe_3_O_4_/HA mixture because of the good dispersion of Fe_3_O_4_ nanoparticles in the HA matrix. There is some concern that HA in the composites can reduce the hyperthermia effect of Fe_3_O_4_ nanoparticles. However, our composites showed good hyperthermia-related properties, which were similar to or better than those reported elsewhere for these materials [22–24].

## 3. Experimental Section

In the experiments, analytical grade reagents (purchased from Wako Pure Chemical Industries, Osaka, Japan) were used as received without further purification. The Fe_3_O_4_ nanoparticles were synthesized by a coprecipitation method reported previously [32]. One millimole of FeCl_2_·4H_2_O and 2 mmol of FeCl_3_·6H_2_O were dissolved in 40 mL of deionized and deoxygenated water. Then, 20 mL of 1.0 kmol/m^3^ NaOH solution was added to the solution at room temperature under vigorous stirring, using a magnetic stirrer, in an argon atmosphere. Immediately after the addition of the NaOH solution (*i.e.*, without aging), the suspension of Fe_3_O_4_ nanoparticles (3.86 mg-Fe_3_O_4_/mL) was diluted with an appropriate amount of deionized and deoxygenated water to adjust the Fe_3_O_4_ concentration in the Fe_3_O_4_/HA nanocomposites. Thereafter, 3 mmol of CaHPO_4_·2H_2_O (DCPD) and 2 mmol of CaCO_3_ (pure calcite), corresponding to the stoichiometric molar ratio in the formation reaction of HA given by Equation (1), were added to 60-mL Fe_3_O_4_ suspensions with variable concentrations (0.44–3.59 mg-Fe_3_O_4_/mL).

After the pH was adjusted with the addition of NaOH solution, the resulting suspension was subjected to a mechanochemical treatment using a horizontal tumbling ball mill. The suspension was placed in a Teflon-lined gas-tight milling pot (inner diameter of 90 mm, capacity of 500 mL). The milling media consisted of commercially available carbon steel balls (SWCH10R, Japanese Industrial Standards (JIS) G 3539; Fe > 99.19 mass%, Mn < 0.60 mass%, C < 0.13 mass%, P < 0.04 mass%, S < 0.04 mass%) with a diameter of 3 mm. The charged volume of the balls (which includes the voids among balls) was 40% of the pot capacity. To prevent the oxidation of Fe_3_O_4_ during milling, the initial oxygen content of the gas phase in the pot was reduced to <2 vol% by introducing argon gas prior to milling. Wet milling was then performed at room temperature for a designated period of time during which the rotational speed of the pot was 140 rpm, corresponding to the theoretically determined critical rotational speed. The pH and milling time were varied over several experiments over the ranges of 12.0–13.5 and 0.5–6 h, respectively. After milling, the precipitate from the pot was washed with deionized water and centrifuged. This washing operation was repeated several times. Lastly, the precipitate was dried overnight at 303 K under vacuum. We also performed an experiment in which the suspension of DCPD and CaCO_3_ was stirred vigorously at room temperature using a magnetic stirrer instead of milling to investigate the effect of milling, *i.e.*, the mechanochemical effect, on the formation of HA.

Powder XRD patterns of our samples were obtained using an X-ray diffractometer (RINT-1500, Rigaku, Tokyo, Japan; CuKα radiation, 40 kV, 80 mA, scanning rate: 1.0°/min). The morphology of the samples was also observed via field emission scanning electron microscopy (FE-SEM; JSM-6700F, JEOL, Tokyo, Japan). The SEM observations were performed at an acceleration voltage of 10 kV after sputter coating the samples with Pt-Pd for 1 min prior to imaging. The particle size distributions were determined using a DLS analyzer (Zetasizer Nano ZS, Malvern Instruments, Worcestershire, UK). The samples were dispersed in deionized water under ultrasonic irradiation before the measurements.

The infrared absorption spectrum of an HA sample was measured with a FT-IR spectrometer (FT/IR-410, JASCO, Tokyo, Japan) in diffuse reflectance mode after the sample was diluted with spectroscopic grade KBr powder in a mass ratio of 1:100. We verified the Ca/P molar ratio using ICP-OES (SPS7800, SII NanoTechnology, Chiba, Japan) for the sample dissolved in a dilute HCl solution. Thermogravimetric analysis was performed using a thermal analyzer (SDT2960, TA Instrument, New Castle, PA, USA) with an argon flow rate of 100 mL/min, where the temperature was increased from ambient to 1173 K at a rate of 10 K/min. The content of carbonate ions was determined by measuring the carbon content through thermal decomposition at 1223 K using a CHN elemental analyzer (2400 Series II, PerkinElmer, Waltham, MA, USA).

For the Fe_3_O_4_ nanoparticles, the magnetic properties were also characterized using a superconducting quantum interference device (SQUID) magnetometer (Quantum Design model MPMS, San Diego, CA, USA).

The magnetic hyperthermia-related properties of our Fe_3_O_4_/HA composites were evaluated using an apparatus that has been described elsewhere [46]. The apparatus mainly consists of a coil for generating an alternating magnetic field, a power supply (radiofrequency power device), and an impedance tuner (matching device). An alternating magnetic field was generated with the use of an external coil comprising 19-turned loops (diameter 65 mm, length 100 mm) of copper pipe (diameter 5 mm) cooled by water to ensure a constant temperature and impedance. The coil was connected to the power supply (T162-5723BHE, Thamway, Fuji, Japan) through the impedance tuner (T020-5723AHE, Thamway, Fuji, Japan). An amount of composite powder was placed in a polystyrene tube (diameter 16 mm) and closely packed by tapping the tube. The loading mass was adjusted such that the packed volume was a constant 0.7 cm^3^, regardless of the Fe_3_O_4_ concentration. We then inserted the tube into the center of the coil and measured the temperature increase of the sample material in an alternating magnetic field using an optical fiber thermometer. The frequency and amplitude of the field were 600 kHz and 2.9 kA/m, respectively. As a control experiment to investigate the milling effect on the magnetic hyperthermia-related properties, the temperature profile of a mixture of Fe_3_O_4_ (10 mass%) and HA was also measured, which was obtained by vigorously stirring the suspensions of Fe_3_O_4_ and HA after they were individually prepared according to the above-mentioned methods.

## 4. Conclusions

A simple mechanochemical method for the rapid synthesis of Fe_3_O_4_/HA composites was developed. In this method, superparamagnetic Fe_3_O_4_ nanoparticles and submicron-sized HA particles are sequentially prepared in a short period at room temperature, and Fe_3_O_4_ nanoparticles are effectively incorporated into the HA matrix by milling. In this study, the milling time required to obtain Fe_3_O_4_/HA composites was only 1 h. In the Fe_3_O_4_/HA composites thus synthesized, the Fe_3_O_4_ nanoparticles were observed to be homogeneously dispersed without having formed any large aggregates, even in the absence of anti-agglomeration agents, illustrating the success of the milling process. This property resulted in effective heat generation in the Fe_3_O_4_/HA composites when the composites were immersed in an alternating magnetic field. The HA powder synthesized by our method was determined to be low-crystalline B-type carbonate HA, which is suitable to serve as a bone-substitute material. Therefore, our synthesis method can efficiently provide Fe_3_O_4_/HA composites that may be used in hyperthermia therapy against malignant bone tumors. Future work will focus on the improvement of the hyperthermia-related properties of the material by Fe_3_O_4_ nanoparticle size optimization and also on applications of the materials in bone tissue engineering.
